# A combination hydrogel microparticle-based vaccine prevents type 1 diabetes in non-obese diabetic mice

**DOI:** 10.1038/srep13155

**Published:** 2015-08-17

**Authors:** Young Mee Yoon, Jamal S. Lewis, Matthew R. Carstens, Martha Campbell-Thompson, Clive H. Wasserfall, Mark A. Atkinson, Benjamin G. Keselowsky

**Affiliations:** 1 Department of Pathology, Immunology and Laboratory Medicine, College of Medicine, University of Florida, Gainesville, FL 32611, USA; 2 J. Crayton Pruitt Family Department of Biomedical Engineering, College of Engineering, University of Florida, Gainesville, FL 32611, USA

## Abstract

Targeted delivery of self-antigens to the immune system in a mode that stimulates a tolerance-inducing pathway has proven difficult. To address this hurdle, we developed a vaccine based-approach comprised of two synthetic controlled-release biomaterials, poly(lactide-co-glycolide; PLGA) microparticles (MPs) encapsulating denatured insulin (key self-antigen in type 1 diabetes; T1D), and PuraMatrix^TM^ peptide hydrogel containing granulocyte macrophage colony-stimulating factor (GM-CSF) and CpG ODN1826 (CpG), which were included as vaccine adjuvants to recruit and activate immune cells. Although CpG is normally considered pro-inflammatory, it also has anti-inflammatory effects, including enhancing IL-10 production. Three subcutaneous administrations of this hydrogel (GM-CSF/CpG)/insulin-MP vaccine protected 40% of NOD mice from T1D. In contrast, all control mice became diabetic. *In vitro* studies indicate CpG stimulation increased IL-10 production, as a potential mechanism. Multiple subcutaneous injections of the insulin containing formulation resulted in formation of granulomas, which resolved by 28 weeks. Histological analysis of these granulomas indicated infiltration of a diverse cadre of immune cells, with characteristics reminiscent of a tertiary lymphoid organ, suggesting the creation of a microenvironment to recruit and educate immune cells. These results demonstrate the feasibility of this injectable hydrogel/MP based vaccine system to prevent T1D.

Type 1 diabetes (T1D) is a chronic autoimmune disorder, characterized by autoreactive T cell-mediated destruction of insulin-producing β-cells in the pancreatic islets of Langerhans^1^. Lack of insulin production leads to aberrant glucose metabolism, hyperglycemia, and ketoacidosis^2^. Moreover, poorly managed blood sugar levels can lead to life-threatening complications including cardiomyopathy, neuropathy, retinopathy, and nephropathy^3^. Although genetic and environmental factors are thought to contribute to the development of T1D, aberrant autoimmune responses are considered the primary insult in T1D initiation and progression^4^. In particular, islet-specific autoantigen-recognizing T cells are believed to play a critical role in this disease. Therefore, autoantigen-specific immunomodulation approaches have gained widespread attention as a preferable and targeted way to prevent or intervene in T1D^5^.

One of the challenging requirements for a successful immunomodulatory vaccine is antigen selection and delivery. In general, soluble antigen tends to be rapidly cleared by the immune system prior to inducing the desired immune responses. Thus, the development of sustained-release synthetic biomaterials has been explored in vaccine design^6^,^7^,^8^,^9^. Tolerance-inducing vaccines have been proposed that aim to reeducate the immune system to a specific autoantigen. Antigen presenting cells are capable of internalizing particulate vaccines, and a possible mechanism of antigen-specific tolerance induction could be via inducing anti-inflammatory cytokine production, antigen-specific T cell anergy, deletion, or regulatory T cell population^10^,^11^,^12^,^13^. Toward that goal, antigen can be incorporated in phagocytosable MPs in order to be taken up by antigen presenting cells. Particles made of PLGA from nanometer to micron sizes have been widely explored for improved delivery of DNA, adjuvant, or antigen to the immune system^6^,^14^,^15^,^16^,^17^. In this study, we utilized PLGA MPs encapsulating insulin, a well-known autoantigen in T1D^2^. In addition, injectable PuraMatrix^TM^ peptide hydrogel was used as a chemokine/adjuvant depot to promote infiltration and immune modulation of lymphocytes, co-localized with antigen-loaded MPs. The PuraMatrix^TM^ hydrogel is a biocompatible and biodegradable self-assembling peptide-based material, which can form a stable 3D structure in the presence of physiological levels of salt, with pore sizes between 5–200 nm in diameter^18^,^19^,^20^, with demonstrated ability to serve as a delivery vehicle for bioactive molecules^21^,^22^,^23^.

Adjuvant choice represents a major obstacle to eliciting desired modulation of adaptive immune responses^24^. Tolerance inducing adjuvants have not been widely explored. CpG oligodeoxynucleotides (ODNs) are synthetic DNA, containing one or more CpG motifs, which are recognized by Toll ligand receptor 9 (TLR9) and can induce strong immune responses safely^25^. It has been most recognized that CpG can trigger T helper type-1 responses by stimulating macrophages, dendritic cells and monocytes to secrete pro-inflammatory cytokines such as TNF-α, IL-1, IL-6, and IL-12^26^,^27^,^28^,^29^, which has supported investigations as an adjuvant in cancer and infectious disease mouse models^30^,^31^. However, several studies indicate that CpG has an ability to boost anti-inflammatory effects through inducing IL-10 secretion, FoxP3+ regulatory T cells in animal models for autoimmune arthritis and T1D^32^,^33^. Along these lines, Mellor *et al*., also showed intravenous CpG induced an immunoregulatory enzyme indoleamine 2,3-dioxygenase in splenic CD19+ dendritic cells in B6 mice and suppressed T cell stimulatory functions^34^. An additional adjuvant, GM-CSF, was utilized as a chemoattractant, since it has the ability to promote recruitment of circulating monocytes, lymphocytes, and neutrophils. This cytokine also can activate and increase the function of antigen presenting cells specifically dendritic cell and macrophage populations^35^,^36^. Multi-component adjuvants combined with biomaterial controlled release platforms represent a promising approach to tailoring tolerance-promoting immune responses. Herein, we formulated a combination hydrogel MP vaccine and investigated prevention of T1D in non-obese diabetic (NOD) mice.

## Methods

### Preparation of human denatured insulin-encapsulated PLGA MP

To avoid metabolic activity of the encapsulated insulin, denatured insulin was used. Recombinant human insulin powder (SAFC Biosciences, Inc., Lenexa, KS, USA) was dissolved in sterile water and adjusted to pH 2.5 with 0.1 N HCl. 10 mM of 2-mercaptoethanol (Sigma-Aldrich, St. Louis, MO, USA) was then added to the insulin solution in order to reduce disulfide bonds, followed by incubation of the solution at 95 ^°^C for 5 min for further denaturing. The heated solution was cooled on ice and 1 N NaOH was added to the solution for pH neutralization, followed by filtration (Amicon Ultra Centrifugal Filter Unit; Millipore, Billerica, MA, USA). Final concentration was measured by Bradford assay (BioRad, Hercules, CA, USA). PLGA MPs were prepared using a standard water-oil-water double emulsion solvent evaporation technique. A 50:50 polymer composition of PLGA (average MW ~44,000 g/mol in methylene chloride; Purac, Lenexa, KS, USA) was used to generate MPs. Poly-vinyl alcohol (PVA) (MW ~100,000 g/mol; Fisher Scientific, Waltham, MA, USA) was used as an emulsion stabilizer. Distilled water (DiH_2_O) was used as the aqueous phase to form the emulsions, while methylene chloride (Fisher Scientific, Waltham, MA, USA) was used as the organic solvent to dissolve PLGA polymer. To incorporate the insulin into the PLGA MPs, 100 mg of PLGA polymer was dissolved in methylene chloride at 5% w/v ratio. Then, 0.1 mL of the denatured insulin solution (1 mg/mL) was added to the 5% PLGA solution and homogenized at 35,000 rpm for 120 s to form a primary emulsion. This emulsion was added to 2 mL of 5% PVA solution and homogenized again at 19,500 rpm for 60 s to form the secondary emulsion which was transferred to a beaker containing 30 mL of 1% PVA. The particles thus formed were agitated using a magnetic stirrer for 24 h to evaporate residual methylene chloride. The remaining solution was centrifuged at 10,000 g for 10 min to collect MPs, which were subsequently washed three times with DiH_2_O. The water was aspirated from the centrifuged MPs, which were then flash-frozen in liquid nitrogen and kept under vacuum in dry ice overnight. The MPs were stored at −20 ^°^C until use.

### PLGA MP characterization

The size distributions of MP were measured using the Microtrac Nanotrac Dynamic Light Scattering Particle Analyser (Microtrac, Montgomery, PA, USA). The MP diameter is reported as mean ± standard deviation. The loading amount of denatured insulin in MPs was measured using a solvent extraction technique followed by spectrophotometric analysis.

### Preparation of PuraMatrix^TM^ peptide hydrogel

The PuraMatrix^TM^ peptide is provided from the manufacturer (BD Biosciences, San Jose, CA, USA) as a 1.0% (w/v) solution at pH 2.0–2.5. In order to help stabilize the hydrogel-incorporated components in acidic pH, the peptide solution was mixed with 10% sterile sucrose (Sigma-Aldrich, St. Louis, MO, USA). For experimental groups which included MPs, a desired mass of the lyophilized MPs was then mixed with the peptide solution. Stock solutions of recombinant mouse GM-CSF (R&D systems, Minneapolis, MN, USA) in sterile PBS (20 ng/μL) and CpG ODN1826 (InvivoGen, San Diego, CA, USA) in endotoxin-free water (5 μg/μL) were prepared. Depending on the experiment, different volumes of these solutions were mixed with the PuraMatrix^TM^ peptide solution to provide desired final concentrations. Once combined, salt ions provided by the PBS caused gelation within 10 min. Formulations were therefore used immediately upon mixing.

### Scanning electron microscope (SEM) imaging

Hydrogels and particle-loaded hydrogels were characterized by a scanning electron microscope (Hitachi S4000, Interdisciplinary Center for Biotechnology Research Core Electron Microscopy Facility, University of Florida). For these studies, a total volume of 100 μL of PuraMatrix^TM^ hydrogel was mixed with 50 μg of PLGA MPs and deposited on glass coverslips. The hydrogels were allowed to crosslink in a 37 °C incubator for 1 h. Afterward, hydrogels were fixed with 4% paraformaldehyde for 30 min followed by washing with PBS. A graded dehydration protocol was then performed. Briefly, solutions of 30, 50, 70, 85, 90, 95, and 100% ethanol in dH_2_O were made. Hydrogels were then incubated in each solution for 15 min. Solutions of 25, 50, 75, and 100% hexamethyldisilazane (HMDS) in 100% ethanol were made. After the final ethanol incubation, samples were incubated in increasing HMDS solutions for 30 min each. A final incubation in 100% HMDS was repeated and samples were then left in a fume hood to dry overnight. Coverslips with dehydrated hydrogels were then mounted for SEM and coated with Au/Pd before imaging.

### *In vitro* hydrogel release assay

To determine the release profiles of murine GM-CSF (80 ng/mL) and CpG ODN1826 (45 μg/mL) from the peptide hydrogel, each agent was incorporated into hydrogel as described above. The total volume of the mixture was 100 μL. Triplicate samples were plated in each well of a 12-well cell culture plate. After the gelation was confirmed, PBS was added into each well followed by placement into a 37 ^°^C CO_2_ incubator. Supernatants were collected and the same volume of fresh PBS was replenished at each time point (0, 0.5, 1, 3, 6, 12, 18, 24, and 48 h). The *in vitro* release kinetics of GM-CSF or CpG from the hydrogel was measured using a murine GM-CSF ELISA kit (eBioscience, Inc., San Diego, CA, USA) or NanoVue Plus^TM^ Spectrophotometer (GE Healthcare Life Sciences, Pittsburgh, PA, USA), respectively.

### Spleen and bone marrow cell purification

All experimental procedures using NOD mice (The Jackson Laboratories, Bar Harbor, ME, USA) were performed in accordance with protocols approved by the University of Florida Institutional Animal Care and Use Committee. After sacrificing mice (female, 8 weeks of age), spleens were collected using sterile surgical instruments and then transferred into a conical tube containing 5 mL Hank’s Buffered Salt Solution (HBSS; Mediatech, Manassas, VA, USA). For splenocyte isolation, the spleen was placed in a sterile 40 μm nylon cell strainer on top of a new 50 mL conical tube and perfused by an HBSS-filled 3 mL syringe-attached 27-gauge needle. Then, the plunger was removed from the syringe and used to crush the spleen. HBSS was added during the tissue grinding until the total volume reached 30 mL. Centrifugation at 300 g for 10 min at room temperature was performed to obtain a cell pellet. The supernatant was decanted and the pellet was resuspended with 2 mL HBSS.

For bone marrow cell purification, the femurs and tibiae from hind limbs were obtained. Attached soft tissues and muscles were carefully removed by using a sterile scalpel. Then, the bones were placed in a 1.5 mL microcentrifuge tube containing 1 mL HBSS. Both ends of the bone were removed using a sterile scalpel and the marrow was flushed by an HBSS-filled 3 mL syringe-attached 27-gauge needle. All isolated marrows were collected into sterile 15 mL conical tubes. Centrifugation at 300 g for 10 min at room temperature was performed to pellet the cells. The supernatant was decanted and the pellet was resuspended in 10 mL HBSS followed by filtration through a sterile 100 μm nylon cell strainer. The cells were then centrifuged at 300 g for 10 min at room temperature. The supernatant was carefully removed followed by resuspension of the cell pellet in 2 mL HBSS.

The following steps were identical for both the spleen and bone marrow cell purification, unless otherwise stated. To lyse the red blood cells, 3 mL of 1x BD Pharm Lyse^TM^ lysing buffer (BD Biosciences, San Jose, CA, USA) were added the tube and placed on ice for 5 min. Two additional washes and centrifugations with 30 mL HBSS were performed. Washed cells were resuspended in 30 mL HBSS for splenocytes and 20 mL HBSS for bone marrow cells. Following this step, the cells were ready for cell counting and further analysis.

### Boyden chamber migration assay

Three NOD mice were sacrificed and then their bone marrow cells were isolated for a Boyden chamber migration assay. Three million whole bone marrow cells in 300 μL RPMI1640 (Mediatech, Manassas, VA, USA) cell culture medium containing 10% fetal bovine serum (Atlanta Biologicals, Flowery Branch, GA, USA) and 1% penicillin/streptomycin (Mediatech, Manassas, VA, USA) were placed in the upper chamber of a sterile 8 μm polycarbonate Millicell^®^ cell culture insert (Millipore, Billerica, MA, USA). The individual inserts were placed in each well of 12-well cell culture plate. Each lower chamber was contained: the cell culture medium alone; 50 μL hydrogel with 0.5 mg empty MPs; 50 μL hydrogel with 0.5 mg empty MPs and 200 ng/mL GM-CSF; or soluble form GM-CSF (200 ng/mL). Each bottom chamber was filled with 500 μL of the cell culture medium. The cell culture plate was placed in 37 ^°^C CO_2_ incubator for 24 h. After this incubation, migrated cells were collected and the number of live cells were counted.

### *In vitro* adjuvant stimulations

To determine the effect of adjuvant stimulations, three NOD mice were sacrificed and the spleen was excised in order to purify splenoctyes. Adjuvant was resuspended in the RPMI1640 cell culture medium for this experiment. *In vitro* stimulation settings were as follows: no stimulant (the cell culture medium alone); CpG (20 μg/mL); GM-CSF (200 ng/mL) and CpG (20 μg/mL). Isolated splenocytes were plated at 1 × 10^5^ cells per well in a round bottom 96-well cell culture plate. The cell culture plate was placed in 37 ^°^C CO_2_ incubator. Supernatants were carefully collected after 24 and 48 h and stored at −20 ^°^C until use. The levels of IL-10 in supernatants were measured by ELISA (eBioscience, Inc., San Diego, CA, USA).

### Diabetes prevention study

Female NOD mice were purchased from the Jackson Laboratories (Bar Harbor, ME, USA) and maintained in specific pathogen free facilities at the University of Florida. All animal experimental procedures were conducted in accordance with protocols approved by the University of Florida Institutional Animal Care and Use Committee. A cohort of 8-week old female NOD mice was divided into two treatment groups (n = 10/group). Experimental groups were as follows: Hydrogel (GM-CSF/CpG) + insulin MPs; Hydrogel (GM-CSF) + empty MPs; and a no treatment control. The vaccine formulations were administered at 8, 10, and 12 weeks of age. Per injection, a total volume of 100 μL was administered, providing the following doses of each component: GM-CSF (40 ng/injection); CpG (10 μg/injection); PLGA MPs (5 mg/injection); 1% hydrogel solution (91 μL/injection). Vaccine formulations were injected at the subcutaneous dorsal neck region. Blood glucose levels were measured once per week with a OneTouch^®^ Ultra^®^ 2 blood glucose meter (Lifescan, Inc., Milpitas, CA, USA). If the value was higher than 250 mg/dL for two consecutive days, the animal was considered to be diabetic and withdrawn from the study.

### Histology and immunohistochemistry of granulomas

Granulomas were excised from the dorsal skin region one week after the third injection (i.e., 13 weeks of age) and fixed in 10% formalin solution overnight at room temperature. Fixed tissues were processed to paraffin blocks by the Molecular Pathology Core at the University of Florida. Paraffin sections (4 μm) were stained with H&E, or by immunohistochemistry with the following primary antibodies: anti- CD45R/B220, clone RA3-6B2 (BD Biosciences, San Jose, CA, USA), anti- CD3, clone CD3-12 (Serotec, Raleigh, NC, USA), anti- F4/80, clone BM8 (Caltag Medsystems, Buckingham, UK), or anti- LYVE-1 (Lymphatic vessel endothelial hyaluronan receptor-1; Abcam, Cambridge, MA, USA). Deparaffinized sections were hydrated then blocked in normal goat serum followed by incubation in primary antibodies for one hour at room temperature. Following washes, sections were incubated with goat anti-rabbit HRP-polymer labeled antibody (Mach2 HRP, Biocare, Concord, CA, USA) for 30 min at room temperature followed by horseradish peroxidase- DAB and hematoxylin counterstain.

### Statistical analysis

Data analyses were performed using GraphPad Prism^®^ v5.0 software (La Jolla, CA, USA). Kaplan-Meier survival curves present diabetes incidence over the time course. Comparing survival curves by Mantel-Cox test determined the p-value and significance. Other data were analyzed by one-way ANOVA with Bonferroni’s multiple comparison tests. Statistical significance was defined if the p-value was less than 0.05. Error bars represented mean ± standard error of the mean (SEM), unless otherwise indicated.

## Results

### Materials, loading and release characterization

**Figure 1:**
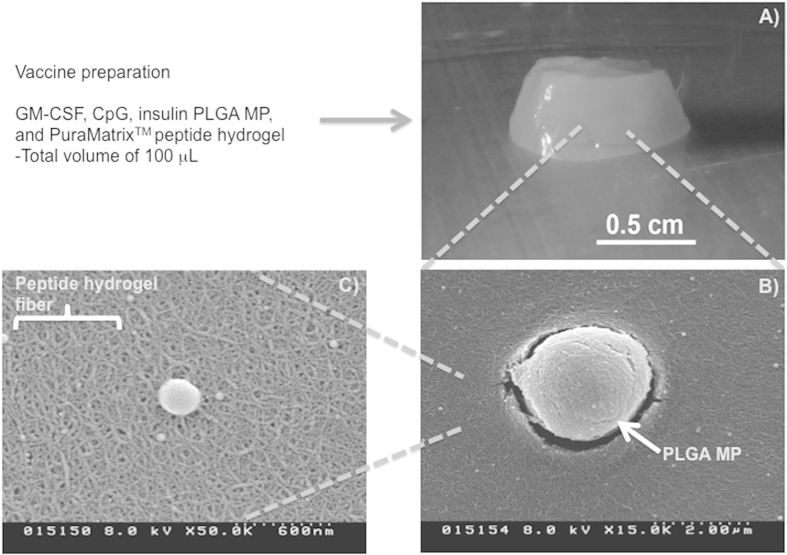
Combination PuraMatrix^TM^ peptide hydrogel/PLGA microparticle (MP) vaccine system. (**A**) A total volume of 100 μL of hydrogel/MP readily formed a ~1 cm diameter x 0.5 cm high 3D hydrogel mixture *in vitro*. Scanning electron microscopy revealed (**B**) the MPs were well-incorporated and (**C**) the hydrogel formed a nano-scale fibrillar network.

**Figure 2:**
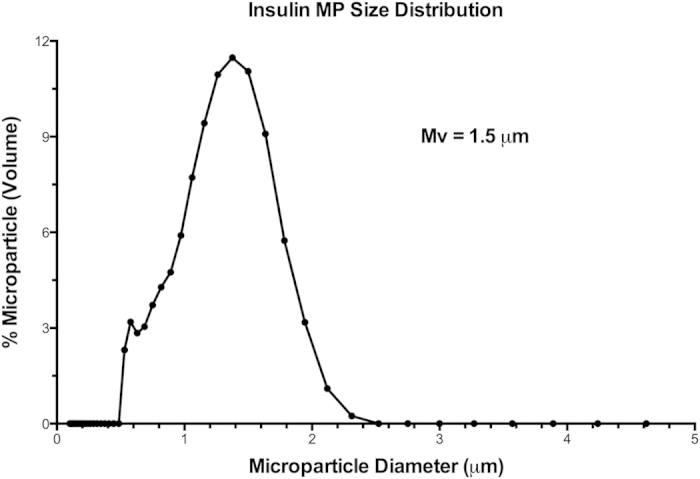
PLGA microparticle (MP) characterization. Insulin MP size distribution was confirmed by dynamic light scattering particle size analysis. Average diameter of the MP was 1.5 μm (by volume-based method).

Both the PuraMatrix^TM^ hydrogel and PLGA MPs have each separately been extensively tested for their ability to function as drug delivery vehicles by other groups in numerous different settings^6^,^9^,^14^,^15^,^16^,^17^,^21^,^37^. To validate the compatibility of the two materials, and verify their ability to form a 3D hydrogel when combined, PLGA MPs were added to PuraMatrix^TM^ and incubated for 1 h to promote gelation (Fig. 1A). SEM imaging showed the surface of the scaffold and confirmed that the MPs were well incorporated into the hydrogel (Fig. 1B). In addition, hydrogel formation was unaffected by the addition of PLGA MPs, demonstrating a fibrous network structure (Fig. 1C). Next, the individual biomaterial components were characterized. First, MPs encapsulating denatured human insulin were observed to have an average hydrodynamic diameter of 1.5 μm, as determined by Dynamic Light Scattering Particle Analyser (Fig. 2). To avoid metabolic activity of the encapsulated insulin, denatured insulin was used. The loading amount of denatured insulin in the MPs was found to be 4 ± 0.3 μg per mg of PLGA MPs.

**Figure 3:**
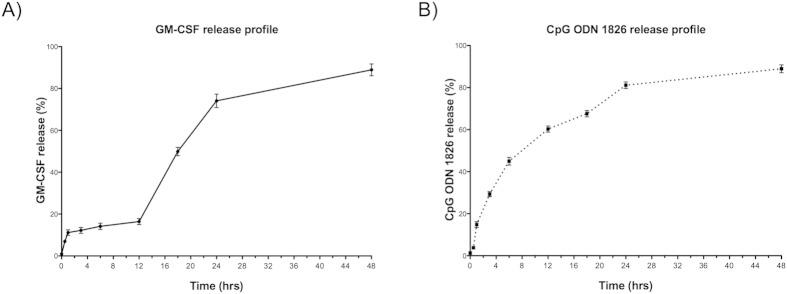
Release profiles of GM-CSF and CpG ODN1826 from the hydrogel. (**A**) Initial release of GM-CSF (80 ng/mL) was delayed for 12 h, followed by a more rapid rate through 24 h, and then a slower release rate through 48 h. (**B**) Rapid initial release of CpG was detected within 12 h, followed by a slower release rate through 48 h. Released amounts of both factors reached almost 90% within 48 h.

**Figure 4:**
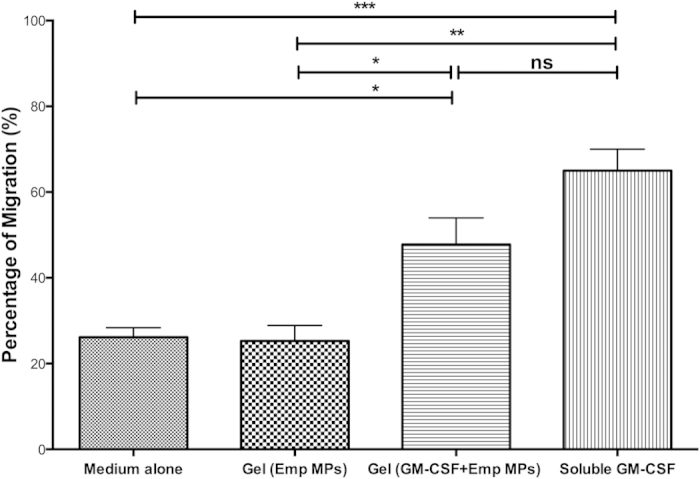
Bioactivity of hydrogel-released GM-CSF as a chemoattractant. Boyden chamber assay revealed that the hydrogel-released GM-CSF was capable of recruiting NOD mouse bone marrow cells (n = 3). Recruited cell numbers were statistically the same as soluble GM-CSF (200 ng/mL) control.

In order to examine the release profile of GM-CSF and CpG from the hydrogel, *in vitro* release was quantified. After 24 h, 80% of the total GM-CSF loaded into the hydrogel had been released (Fig. 3A). In contrast, CpG was released from the hydrogel at a higher rate, indicated by 60% cumulative release after just 12 h (Fig. 3B). This is consistent with the fact that the molecular weight of GM-CSF (14.3 kDa) is considerably higher than CpG (6.6 kDa). After 48 h, both agents were almost completely released from the hydrogel. To determine biological activity of cytokine released from the hydrogel, an *in vitro* Boyden chamber migration assay was performed. Hydrogel loaded with GM-CSF and empty MPs served as the experimental group, while soluble GM-CSF was the positive control. As negative controls, unloaded hydrogel plus empty MPs, as well as media alone, were used. Bone marrow cells were significantly mobilized both by soluble GM-CSF (p < 0.001) and hydrogel-released GM-CSF (p < 0.05) compared to controls. There was no statistical difference in migration between soluble and hydrogel-released GM-CSF (Fig. 4). This result indicates that the hydrogel encapsulation and release process did not affect biological function of GM-CSF, which successfully promotes chemotactic recruitment of bone marrow derived immune cells.

### Type 1 diabetes prevention

In order to determine the combination hydrogel/MP vaccine efficacy for preventing T1D, vaccine formulations were subcutaneously injected in 8-week old female NOD mice (n ≥ 10/group), with two additional injections given at 10 and 12 weeks of age as a booster. NOD mice at 8 weeks of age typically undergo progressive peri-insulitis, with the production of anti-insulin autoantibodies detectable^2^,^4^,^5^. Diabetes incidence was tracked over ~5 months, until the mice were 28 weeks old. Dosing amounts of each factor per injection were calculated to be 20 ± 1.5 μg of denatured insulin encapsulated in MPs, and 40 ng of GM-CSF and 10 μg of CpG incorporated in the hydrogel (Table 1). For the hydrogel-incorporated factors, losses were considered negligible given that the processing is minimal and entirely aqueous based. The full vaccine formulation group (Hydrogel (GM-CSF/CpG) + Insulin MPs) was compared to a no treatment control, as well as a GM-CSF-only group (Hydrogel (GM-CSF) + Empty MPs).

**Figure 5:**
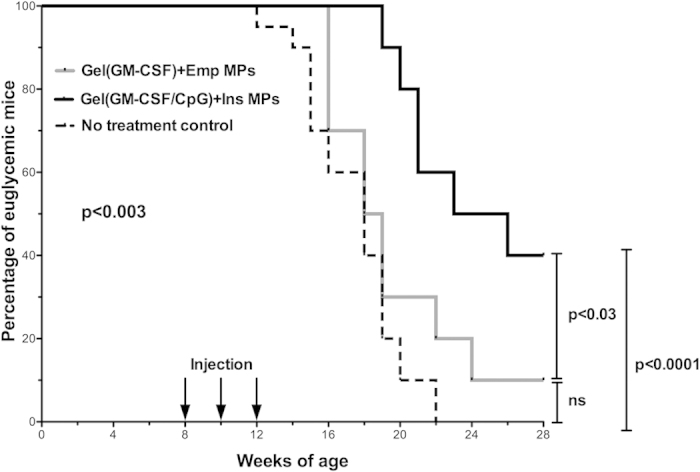
Hydrogel/microparticle vaccine prevents type 1 diabetes in NOD mice. Kaplan-Meier plots demonstrate the Hydrogel (GM-CSF/CpG) + Ins MP vaccine (n = 10) provided protection from diabetes in 40% of the cohort, with a delayed onset to 19 weeks. In comparison, the no treatment control group (n = 20) showed 0% of the cohort remained diabetes free, and onset began at 12 weeks of age. Additionally, the GM-CSF only control, Hydrogel (GM-CSF) + Empty MP (n = 10), performed no better than the no treatment group. (Overall p-value: p < 0.003).

Results were plotted using Kaplan-Meier survival curves, showing the fraction of the cohort that were euglycemic (non-diabetic) versus the age of the mice, and the Mantel-Cox test was used to determine significance. Significant differences were found between experimental groups (overall p < 0.003). Onset of diabetes began at 12 weeks of age for the no treatment group (Fig. 5). Notably, onset was delayed to 19 weeks for the mice given the combination hydrogel/MP based vaccine (Hydrogel(GM-CSF/CpG) + Ins MP group). More importantly, whereas all of the mice became diabetic in the no treatment group by 22 weeks of age (0% survival fraction), the survival fraction in the combination vaccine treatment group was significantly improved to being 40% diabetes-free at 28 weeks of age (p < 0.0001). This result demonstrates clear benefit of the combination hydrogel/MP formulation in preventing autoimmune diabetes.

In order to investigate the role of specific factors, the Hydrogel (GM-CSF)+Empty MP group was included, which delivered the same hydrogel controlled release scenario as the full formulation, as well as including unloaded PLGA MPs. For this GM-CSF only group, onset of diabetes was delayed to 16 weeks of age, and the diabetes-free fraction was found to be 10%, which potentially suggested a small improvement over the no treatment control. However, statistical comparison indicated that there is no difference between the GM-CSF only group and the no treatment group (p > 0.12). On the other hand, comparing the GM-CSF only group (Hydrogel (GM-CSF)+Empty MPs) to the full formulation group (Hydrogel (GM-CSF/CpG) + Insulin MPs) yielded statistical significance (p < 0.03). These results suggest that the neither the biomaterials (PuraMatrix^TM^ hydrogel and PLGA MPs) nor the GM-CSF, at the amounts used, were able to provide therapeutic benefit for diabetes prevention except when the hydrogel/MP biomaterials system also simultaneously provided CpG and insulin antigen.

### Cytokine production by adjuvant stimulations *in vitro*

**Figure 6:**
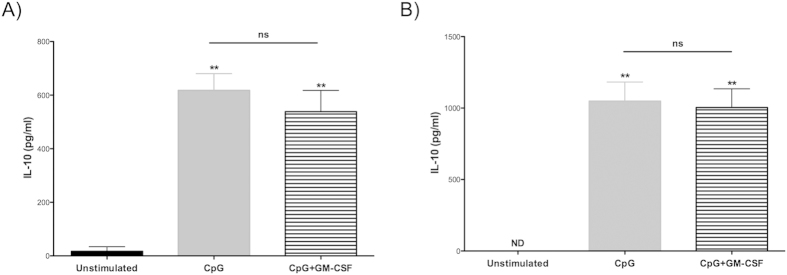
Effect of CpG adjuvant simulation on anti-inflammatory IL-10 cytokine production *in vitro*. Stimulation of CpG induced significantly higher levels of IL-10 production from NOD mice splenocytes (n = 3, p < 0.01). The addition of GM-CSF did not boost IL-10 secretion (**A**); 24 h, and (**B**); 48 h.

In order to examine the effect of the adjuvant stimulation on cytokine production of splenocytes, IL-10 secretion in supernatants was quantified by ELISA. High levels of IL-10 were detected from CpG alone (p < 0.01) and from the CpG plus GM-CSF treated samples (p < 0.01), compared to the untreated samples at both 24 h and 48 h time points post treatment (Fig. 6A,B). Notably, GM-CSF in combination with CpG did not significantly boost IL-10 production compared to CpG alone. Additionally, IL-10 levels were higher at 48 h than at 24 h for both the CpG and the CpG plus GM-CSF-groups.

### Histopathological analysis of granuloma

**Figure 7:**
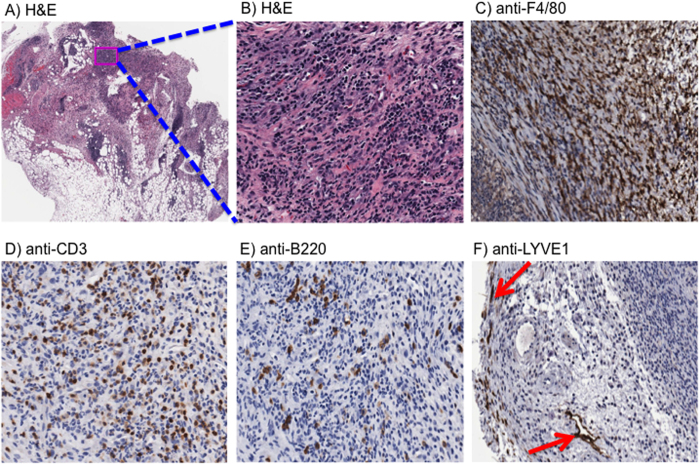
Histopathology of injection sites. Subcutaneous injections of the hydrogel/microparticle vaccine induced granuloma formation at the injection site. (**A,B**) H&E staining of granuloma sections demonstrate proteinaceous structures with high levels of nucleated cell infiltration. Immunohistological staining revealed infiltrating cells consisted of (**C**) macrophages, (**D**) T lymphocytes, and (**E**) B lymphocytes. Additionally, the surrounding area of granuloma exhibited a rare, but positive staining for (**F**) lymphatic endothelial marker LYVE-1.

A week after the second subcutaneous injection, lumps were noticeable upon palpation at the injection site. Upon incision, granuloma-like lesions at the injection site were found in mice that were injected with the full formulation group (Hydrogel (GM-CSF/CpG) + Insulin MPs). In contrast, granulomas could not be found in mice receiving the GM-CSF-only formulation (Hydrogel (GM-CSF) + Empty MPs). Histopathological results of the granulomas showed high levels of proteinaceous deposition with a high degree of nucleated cell infiltration (Fig. 7A,B). Cell phenotypes of the infiltrating cells in the granuloma were characterized. The infiltrates consisted of high numbers of F4/80+ macrophages (Fig. 7C), CD3+ T cells (Fig. 7D), and B220+ B cells (Fig. 7E). Additionally, evidence of rare LYVE-1 staining of a lumen containing structure within the granuloma is shown (Fig. 7F**, arrows**), and the surrounding area of the granuloma was also positively stained with LYVE-1. These features are consistent with a primary granuloma with areas of tertiary lymphoid tissue. Critically, it was noted that the granulomas were resolved by 28 weeks of age, as determined by palpation and surgical examination. Altogether, these characteristics support the concept of the formation of temporary immune educating microenvironment at the subcutaneous injection site where antigen presenting cells and lymphocytes are recruited and educated by the hydrogel/MP vaccine formulation.

## Discussion

Our investigations characterized the capability of a combination of biomaterials, PuraMatrix^TM^ hydrogel and PLGA MP, providing targeting and controlled release of a novel combination of clinically relevant antigen and adjuvants, to prevent T1D in NOD mice. These and related biomaterials have been widely examined as controlled release systems^6^,^15^,^21^,^37^,^38^. For instance, the Langer group delivered antigen via non-degradable biomaterials to immunize animal models in studies conducted more than three decades ago^39^. Advancing this approach, Mooney and colleagues recently demonstrated a promising biomaterial scaffold-based cancer vaccine, which consists of biodegradable PLGA scaffold with GM-CSF, CpG and tumor antigen. This scaffold was subcutaneously implanted in mice in order to recruit and educate immune cells, particularly dendritic cells. As a result, 90% of the vaccine implanted mice survived with increased activation of dendritic cells and lymph node homing of these cell populations, promoting tumor antigen specific T cell populations^40^. Although this “infection-mimicking” cancer vaccine formulation shares adjuvant components with our tolerogenic diabetes vaccine, the end results of respective study were notably different. A number of possible reasons may explain this difference. First, there is a large difference in adjuvant dose (3000 ng GM-CSF and 100 μg CpG^40^, versus 40 ng GM-CSF and 10 μg CpG, as shown in Table 1). Their formulation used porous PLGA scaffold wafers for longer release time (weeks) to provide a prolonged pro-inflammatory environment. In contrast, we incorporated the adjuvants into a hydrogel, providing very short release profiles (hours; Fig. 3). Furthermore, surgical incision is required to implant their scaffold, adding to the inflammatory response. Thus higher adjuvant dose, longer release profile of adjuvants, and the incision for the implantation contribute to their inflammatory “infection-mimicking” system. Conversely, our formulation of a low dose of CpG coupled with insulin antigen and GM-CSF are expected to stimulate transient and subsequently resolved inflammation. The concept of inducing inflammation resolution resulting from a transient inflammatory response has begun to be emphasized as a potential therapeutic target for inflammatory diseases^41^,^42^. The Mooney biomaterial scaffold vaccine, as well as work by the Roy, Shea, Collier, Little and Irvine groups^43^,^44^,^45^,^46^,^47^,^48^,^49^,^50^,^51^, served as inspiration for the present approach, in terms of using biomaterials to provide a controlled microenvironment for immune modulation.

Biomaterials-based immunomodulation is a burgeoning field^52^,^53^,^54^,^55^,^56^,^57^,^58^,^59^,^60^, increasingly gaining attention. In contrast, specific application of biomaterials to autoimmune disease therapies such as T1D has only recently begun to be explored. In particular, two particle vaccine related approaches for the amelioration of T1D have been recently reported^61^,^62^. Santamaria and colleagues used MHC I tetramers coupled to iron oxide nanoparticles to deplete effector cells^61^, while Giannoukakis and colleagues used polymeric microparticles encapsulating anti-sense oligonucleotides for costimulatory molecules to inhibit maturation of antigen presenting cells^62^. These reports, in part, also served to motivate our approach in terms of using synthetic particle-based vaccines for T1D. Furthermore, our approach builds from these concepts, with an approach that has both widespread off-the-shelf potential capability in contrast to the Santamaria approach, which has the challenge of histocompatibility matching the MHC-I complexes for each patient. Our approach has the additional benefit of specificity via delivered antigen, in contrast to the Giannoukakis approach, which is not antigen-specific, and has potential to be systemically suppressive.

The combination hydrogel/MP approach incorporates demonstrated biomaterials strategies to provide a temporary local microenvironment, recruiting and locally conditioning immune cells, while also providing particulate antigen. Each material has already been well defined as a delivery vehicle, but the combination of PLGA MPs and PuraMatrix^TM^ peptide hydrogel has not been investigated as a combined delivery system, to the best of our knowledge. The PuraMatrix™ peptide-based hydrogel is a commercially available synthetic matrix (GMP clinical-grade is available) that is used to create well-defined 3D microenvironments. It consists of peptides (1% w/v and 99% water) from standard amino acids including arginine, alanine, and aspartic acid, so that degradation of the hydrogel does not produce toxic products^37^. In physiological solutions, the peptides self-assemble into a 3D hydrogel that exhibits a nanometer scale β-sheet structure-based fibers with an average pore size of 5–200 nm, capable of encapsulating proteins^18^,^19^,^20^. The PuraMatrix™ hydrogel has been shown to provide a scaffold that supports attachment, growth and function of numerous cell types^63^,^64^,^65^. The soluble material can be injected *in vivo* and will subsequently form a 3D hydrogel upon contact with the physiological environment^21^,^66^.

For amelioration of T1D, the choice of adjuvant is driven by the need to drive the immune response toward tolerance. Traditionally, adjuvants in vaccine development are provided to enhance immunogenicity. Counter-intuitively, such adjuvants have also been successful at ameliorating T1D in animals and have been used in human vaccine trials. As an example, incomplete Freunds adjuvant with B_9–23_ insulin peptide was used in a clinical trial, and mechanistically showed increased antibody response as well as T cell response to insulin^67^. Another example is the clinical trial using the β-cell antigen, GAD65, formulated in alum which showed not only an increase in auto-antibody titer to GAD, but in addition, a slowing in the loss of C-peptide after disease onset^68^. While larger clinical trials did not show efficacy by meeting the primary outcome metric of reversing T1D, no safety concerns were noted^69^. The promising mechanistic secondary outcome results of these trials suggest that the so-called pro-inflammatory adjuvant agents have the potential to yield efficacy for tolerance, but are in need of optimizing.

Motivated by these considerations, we have focused on the use of CpG–the unmethylated linear DNA sequence of cytosine nucleotide phosphate-linked to guanine nucleotide. CpG is currently being widely explored in clinical trials for a range of immunotherapy applications, primarily for cancer. There are four classes of CpG ODNs (A, B, C, and P), based on differences in sequence motifs, palindromic sequences, and biological activities^70^. In particular, B-class CpG has been widely tested for its adjuvant activity in vaccine studies due to its ability to provoke strong immunity, through TLR9 signaling and B cell activation compared to the other classes^70^. CpG is detected by TLR9 on dendritic cells and B cells in humans, and is primarily recognized as pathogenic DNA. TLR9 ligation engages the inflammatory innate pathway of activation through the myeloid differentiation primary response gene 88 and inflammasome signaling^71^. Other adjuvants that have been used, particularly alum^72^, also engage an inflammasome pathway and a number of studies have used these types of adjuvants in mice^73^ and the already mentioned attempts in humans^68^ to prevent or reverse T1D. While potentially counter-intuitive, there is an established track record of using so called inflammatory adjuvants in T1D and immunotherapy for other autoimmune cases. For example, the use of CpG as an adjuvant in allergen-specific immunotherapy in mice is promising^74^.

Granulocyte-macrophage colony-stimulating factor, on the other hand, has been demonstrated to be a potent stimulator of immature dendritic cell recruitment and is marketed under the trade name Leukine for a number of FDA-approved applications^40^,^75^,^76^. GM-CSF is a critical mediator in differentiation and development of myeloid dendritic cells that is effective in low doses; resulting in tolerogenic dendritic cells with low expression of MHC II, co-stimulatory molecules (CD80, CD86), and inflammatory cytokines (IL-1, IL-6, IL-8, TNF-α), and increased expression of PDL1. These dendritic cells have been shown to be capable of inducing regulatory T cells with increased secretion of IL-10^77^,^78^,^79^,^80^. Notably, administration of soluble GM-CSF has previously has been tested for diabetes prevention in NOD mice^78^,^81^,^82^. Gaudreau *et al*., demonstrated that numerous high doses of intraperitoneal GM-CSF treatments (100 ng/mouse, 3 times/week until 6-week old, 2 times/week thereafter until 52-week old), started at the young age of 3-week old could protect NOD mice from diabetes, with signs of tolerogenic dendritic cells and regulatory T cells inductions^81^.

In order to characterize our biomaterial-based vaccine, we initially investigated if the hydrogel could mix with PLGA MPs, and still maintain integrity of the hydrogel fibrillar structure. Analysis by SEM confirmed that the MPs were well incorporated into the hydrogel, and a fibrillar network was formed as expected. Loading and timing of release of the immune modulating factors are also important considerations. While PLGA particles encapsulating insulin have previously been reported for controlled release of soluble, metabolically active insulin^83^, we utilized PLGA MP as a phagocytosable carrier of denatured insulin for antigen depot delivery to antigen presenting cells. The hydrogel release of GM-CSF and CpG over a 1–2 d time span is conceptualized as being appropriate to initially support recruitment and activation of immune cell populations, while subsequently providing a persistent depot of particulate insulin autoantigen in the PLGA MPs. Furthermore, it was established that the GM-CSF release from the hydrogel was at high enough levels to support significantly mobilize NOD mouse bone marrow cells.

In this study’s most important finding, using the NOD mouse model of T1D, *in vivo* efficacy of the hydrogel/MP vaccine formulation was clearly established. Onset of diabetes was delayed from 12 weeks to 20 weeks of age, and the survival fraction improved from 0% of the non-treated, to 40% of the vaccine treated cohort remaining diabetes free. Optimization of the dosing and schedule is of great interest to investigate if the survival fraction can be improved upon in the future. Interestingly, the ability of the GM-CSF only hydrogel/MP formulation to prevent diabetes was no better than the no treatment control, highlighting the importance of the CpG and insulin MP components in the formulation. While Gaudreau *et al*., found GM-CSF was capable of preventing T1D in NOD, mice, their dosing provided 2.5 times more GM-CSF as ours for each administration, and they administered the factor 100 times throughout the study^81^, compared to only a total of 3 injections in our study. These large differences in dose and frequency of GM-CSF administration are obvious reasons for these differing outcomes.

Following *in vivo* validation of the vaccination efficacy, the *in vitro* adjuvant stimulatory capacity of NOD splenocytes was explored. Interestingly, significant amounts of IL-10, an anti-inflammatory cytokine, were detected in the presence of CpG. Although CpG is mainly known as a Th1-biased immunostimulator, which induces inflammatory immune responses^84^, CpG has also been shown an ability to promote the anti-inflammatory cytokine IL-10 in dendritic cells^85^,^86^. A role for IL-10 has been emphasized as a beneficial immunoregulatory cytokine in autoimmune diseases including multiple sclerosis, lupus erythematosus^87^, and T1D^88^,^89^. In fact, successful diabetes interventions in NOD mice has been reported by either exogenous IL-10 treatment^88^,^90^,^91^, or by IL-10 gene delivery^89^. Interestingly, we found that the addition of GM-CSF with CpG did not further boost IL-10 production in splenocytes.

Notably, the hydrogel/MP vaccine induced formation of a temporary granuloma, with infiltration of high levels of T cells, B cells and macrophage populations, and features consistent with a tertiary lymphoid organ. Although more detailed characterization of the granuloma formation is required, this observation suggests that this combination hydrogel/PLGA MP vaccine may create a temporary environment in which high number of immune cells are recruited and potentially be re-educated toward a tolerance promoting axis. Overall, our biomaterial-based vaccine system consisting of an injectable hydrogel with adjuvants and insulin antigen encapsulated PLGA MPs exhibited promising potential for tolerance induction in this T1D model.

## Conclusion

Successful prevention of T1D in NOD mice was achieved following three injections of a vaccine delivery system comprised of hydrogel (GM-CSF/CpG) and insulin MPs. Mechanisms for this result were explored and found that increased IL-10 production by CpG administration was likely a contributing factor. Further, following a second administration of the vaccine formulation, granuloma formation at the injection site was observed which contained temporary foci of tertiary lymphoid tissue. This finding highlights a benefit the use of hydrogel/PLGA MP vaccine delivery system, which can create an *in vivo* artificial environment to recruit and educate immune cells to promote tolerance in autoimmune disease.

## Additional Information

**How to cite this article**: Yoon, Y. M. *et al*. A combination hydrogel microparticle-based vaccine prevents type 1 diabetes in non-obese diabetic mice. *Sci. Rep*. **5**, 13155; doi: 10.1038/srep13155 (2015).

## Figures and Tables

**Table 1:** Vaccine component dosing.

Vaccine component	Injection dose (per injection)
Denatured human insulin encapsulated PLGA MPs	5 mg MPs/20 ± 1.5 μg mass of denatured insulin
GM-CSF	40 ng
CpG ODN 1826	10 μg
